# Nashville Medical School

**Published:** 1844-02

**Authors:** 


### NASHVILLE MEDICAL SCHOOL.

The Trustees of the Nashville University are about to organize a Medical Department in that distinguished institution. We have heard the names of several of our medical friends mentioned in connexion with the various professorships, but, so far as we have heard, no elections have yet been made. Nor, we believe, is the time yet fixed for putting the school into operation. We presume, however, that an announcement by the Trustees may be looked for soon.

